# E-cigarette or vaping product use–associated lung injury outbreak and public perceptions and trends in smoking cessation discussions on Twitter

**DOI:** 10.1371/journal.pone.0332414

**Published:** 2025-09-18

**Authors:** Yuqi Zhang, Yingning Wang

**Affiliations:** 1 Center for Data Science, New York University, New York, New York, United States of America; 2 Institute for Health & Aging, School of Nursing, University of California, San Francisco, San Francisco, California, United States of America; 3 Center for Tobacco Control Research and Education, University of California, San Francisco, San Francisco, California, United States of America; 4 Philip R. Lee Institute for Health Policy Studies, University of California, San Francisco, San Francisco, California, United States of America; University of Saskatchewan, CANADA

## Abstract

**Background:**

Cigarette smokers often use e-cigarettes to quit smoking. The outbreak of e-cigarette, or vaping, product use-associated lung injury (EVALI) in 2019 summer sparked discussions about vaping. However, there is a gap in exploring EVALI’s impact on discussions related to smoking cessation and vaping in smoking cessation on social media.

**Objective:**

This study examines trends, sentiments, and topics in smoking cessation discussions before, during, and after EVALI.

**Methods:**

English tweets from September 1, 2018, to January 31, 2020, were collected using snscrape, filtered for smoking-cessation-related keywords. Sentiments were assessed with Valence Aware Dictionary and sEentiment Reasoner (VADER), categorizing tweets as positive, negative, or neutral. Topics were identified via Latent Semantic Analysis, and LexRank was used to extract representative sentences for qualitative insights into the discussions.

**Results:**

Among 397,528 smoking cessation-related tweets, discussions significantly increased in September 2019, accompanied by a decline in sentiment scores. Five topic groups—“Vaping”, “Cannabis”, “Stop Smoking”, “Gum”, and “Tobacco”—were identified. “Vaping” dominated the entire timespan, surpassing other topics in volume. Sentiment scores decreased for “Vaping”, “Stop Smoking”, and “Cannabis” in September 2019, while remaining stable for “Gum” and “Tobacco”. Representative sentences showed that despite the EVALI outbreak, many individuals still perceived vaping as an effective smoking cessation tool and oppose tobacco control policies targeting e-cigarette flavors.

**Conclusions:**

The results deepen our comprehension of how perceptions regarding smoking cessation evolve during EVALI, offering insights for refining public health communication strategies in future health crises.

## Introduction

Smoking is a leading cause of preventable disease, disability, and death in the United States, accounting for 480,000 deaths annually due to cigarette smoking alone [1]. Although the overall prevalence of cigarette smoking has declined, in 2021, there were an estimated 28.3 million U.S. adults (11.5%) currently smoking cigarettes [2,3]. In pursuit of Healthy People 2030, which aims to reduce adult cigarette smoking prevalence to 6.1% [3], dedicating efforts to smoking cessation remains a steadfast commitment to enhancing public health.

E-cigarette devices heat a liquid solution in a reservoir, producing aerosol that users inhale through a mouthpiece [4]. Although there is a lack of consistent evidence indicating the effectiveness of e-cigarettes in smoking cessation [5,6], over half of adult cigarette smokers in the U.S. used e-cigarettes in their most recent attempt to quit smoking from 2014 to 2016 [7]. E-cigarette, or vaping, product use-associated lung injury (EVALI) emerged in 2019, reaching its cases peak in September of 2019, and resulted in 2,807 hospitalizations and 68 reported deaths [8]. Research has indicated that many individuals altered their perception of vaping risks, considering it more risky than previously believed [9–13]. It prompted the investigation into how the outbreak of EVALI has influenced smoking cessation efforts, given that many cigarette smokers used e-cigarettes to quit.

Despite a study conducting online surveys among ever smokers before and after the EVALI outbreak, finding minimal changes in beliefs about e-cigarettes as smoking cessation aids [13], the survey had limited participants and lacked national representativeness. Consequently, the study’s conclusion may be limited in its generalizability.

Social media platforms offer a wealth of publicly accessible content, allowing individuals to freely express their opinions and share their experiences, including those related to smoking cessation [14]. Twitter, used by 22% of U.S. adults [15], is an important platform for disseminating public health information and shaping readers’ emotions and behaviors [16]. Recognizing social media as a crucial tool for health researchers to assess public sentiments, perceptions, and attitudes about health information, policies, and public health events or crises [17–19], various studies have been designed to understand public discourses in social media like Twitter in response to public health issues and events. A prior study found generally favorable attitudes toward e-cigarettes as cessation aids on Twitter [20], but it was limited to 2014 tweets and did not perform sentiment analysis or topic modeling. Due to its early study period, it also missed examining shifts in sentiment during significant events like EVALI. While other research has noted increased discussions around vaping’s negative health impacts during EVALI [14,21], none have specifically analyzed how this event influenced smoking cessation conversations. To address this gap, the present study will analyze tweets from September 1, 2018, to January 31, 2020, examining changes of discussion volume, sentiments, and topics related to smoking cessation before and after the EVALI outbreak. We hypothesize a decrease in sentiment regarding smoking cessation and vaping in smoking cessation and an elevated number of vaping-related posts in smoking cessation discussion during EVALI compared to the pre-outbreak period. The key insights into public perceptions, attitudes, and concerns regarding smoking cessation and the use of e-cigarettes in smoking cessation from this study can enhance our comprehension of how smoking cessation discussions evolve during EVALI and help improve public health communication efforts in future critical health events.

## Methods

### Data collection

The tweets were collected with snscrape [22],an open-source scraper for social media. The collection procedure is as follows. We first used smoking-cessation-related keywords to search English tweets between September 1, 2018, and January 31, 2020 (both included). The search keywords were selected from those used in the literature [20]: “quit smoking”, “quitting smoking”, “quitted smoking”, “quits smoking”, “stop smoking”, “stopping smoking”, “stopped smoking”, “stops smoking”, “quit cigarettes”, “quitting cigarettes”, “quitted cigarettes”, “quits cigarettes”, “stop cigarettes”, “stopping cigarettes”, “stopped cigarettes”, “stops cigarettes”, “smoke less”, “smoking less”, “smoked less”, “smokes less”, “nrt”, “patch”, “lozenge”, “spray”, “gum”, “nicorette”, “cessation”, “#quit”, “#quitsmoking”, “#quitsmokingcigarettes”, “#Cessationnation”. Tweets were then cleaned by removing email addresses, usernames, and URLs within the text (the final sample size is 397,528). We did not utilize contact information in our analysis. All data collected from Twitter were publicly accessible, and our data collection methods complied with Twitter’s terms of service.

### Temporal analysis and sentiment analysis

We organized tweets by month and calculated their monthly counts. In addition, we employed Valence Aware Dictionary and sEntiment Reasoner (VADER) [23] to measure the sentiment intensity of each tweet. VADER is a sentiment analysis tool that generates a compound sentiment score ranging from −1 to 1 where higher absolute values indicate stronger negative or positive sentiment.

To prevent keywords related to smoking cessation from influencing the sentiment scores of the tweets, we replaced them with a neutral placeholder “X”.

After this adjustment, we applied VADER to analyze the tweets and generate compound scores. Each tweet was then classified as positive, negative, or neutral based on its compound sentiment score ([0.05,1.00] for positive, (−0.05,0.05) for neutral, and [−1.00, −0.05] for negative, as default settings by VADER). Subsequently, we computed the monthly average sentiment score for each category as well as overall.

To validate VADER’s robustness, we compared it with three other models: TextBlob, EmoBart, and Twitter-roBERTa-base. A sample of 10,000 tweets was analyzed using these models. As there were no ground-truth labels for these tweets, we calculated the correlation or the percentage of mutual agreement between VADER’ and other models’ sentiment intensity scores, indicating consistency across models (details and results are shown in S1 Appendix).

### Topic modeling with Latent Semantic Analysis

To incorporate phrases, we created bigrams (two-word phrases) and trigrams (three-word phrases) by concatenating lowercase words using the Natural Language Toolkit (NLTK) package [24]. Additionally, all words were lemmatized using NLTK to mitigate the influence of tense and variants. All tweets were then transformed into a Bag-of-Words dictionary(where we then store the Term Frequency Inverse Document Frequency(TF-IDF) [25] value for every occurring word) to vectorize the tweets.

Latent Semantic Analysis (LSA) was applied to find latent topics within tweets [26,27]. LSA operates on the principle of Singular Vector Decomposition, considering each document (here referring to a tweet) as carrying multiple topics with varying weights, and each topic is composed of numerous terms with different weights. We chose LSA for its high computation speed and straightforward interpretability and used the TruncatedSVD (i.e., LSA) module from the scikit-learn [28] package for the actual implementation. The number of topics was preset to 10, following the approach of Westmaas et al. [29]. To validate our choice of the number of topics, we tested a range of values from 2 to 14 and employed the coherence score to assess the semantic consistency within topics [30]. The coherence score measures semantic similarity between the top words in a topic, based on their co-occurrence in the text. A higher coherence score suggests that the top words in a topic are more likely to co-occur, making the topic more inter-relevant.

For each topic identified through LSA, we selected the ten most significant words based on their weights. We normalized these weights using the softmax function to denote their relative importance within their topics. We manually grouped these topics into more explainable categories according to their semantic meaning. Each tweet was then assigned to only one category based on its largest weight topic. We recalculated the monthly average sentiment score for all categories.

### Text summarization with LexRank

Topic modeling is informative in terms of finding heating topics. Nevertheless, it can not capture how the topics frame discussions. To provide a qualitative understanding of the discussions before, during, and after the peak of EVALI(September 2019), we applied LexRank, a graph-based algorithm to compute the representativeness of textual units and extract representative sentences based on scores [31], which performs well on opinionated texts summary [32]. Due to the limit of computational memory, we selected the top 5 representative sentences every week for the three periods, then performed LexRank again to choose the top 25 distinct sentences among all. Stop-words were removed in this case. Besides, we applied LexRank to obtain ten representative tweets for each topic group.

## Results

### Temporal sentiment trend

**Fig 1:**
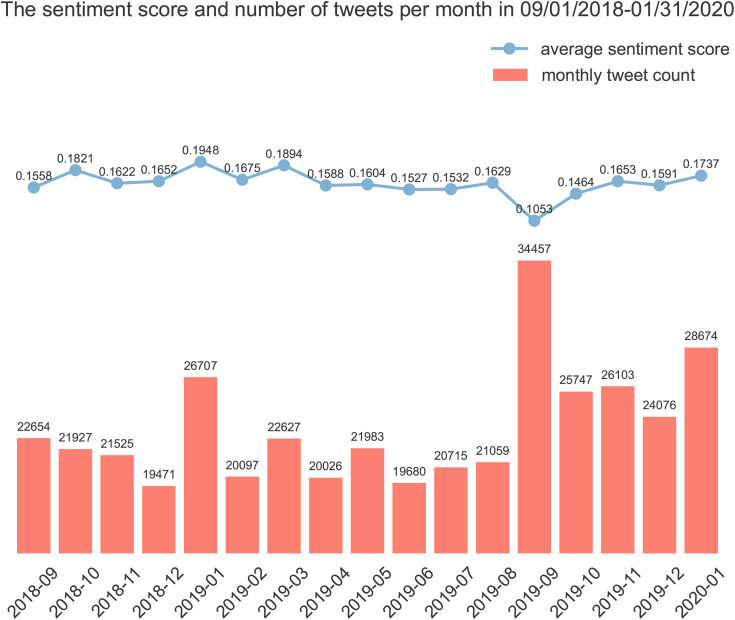
The sentiment score and number of tweets per month from 09/01/2018 to 01/31/2020.

**Fig 2:**
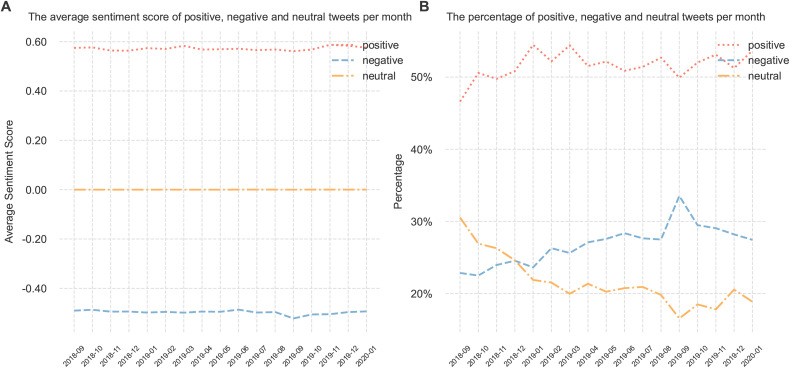
Volume distribution and sentiment of positive, negative, and neutral tweets. (A) The average sentiment score of all tweets per month (B) The percentage of tweets per month.

Table 1 provides representative examples of sentiment intensity measured by VADER, including three examples each for positive, negative, and neutral tweets. By aggregating tweets by their month of creation, Fig 1 displays their average monthly sentiment scores along with the monthly count of smoking cessation-related tweets from September 2018 to January 2020. The monthly tweet count fluctuated throughout the period, with a notable peak of 34,457 tweets in September 2019. Although all sentiment scores remained positive, the sentiment scores of tweets dropped from 0.16 in August 2019 to 0.11 in September 2019, reflecting a decrease in sentiment intensity despite the increased volume of tweets during this period. Excluding September 2019, we observed a positive correlation of 0.309 between the monthly volume of smoking cessation tweets and the average monthly sentiment score, diverging from the variation in September 2019. To explore the underlying factor for this variation, Fig 2A illustrates that the average monthly sentiment scores of positive, negative, and neutral tweets remained relatively stable over time. However, Fig 2B demonstrates a notable change in the proportion of tweets, characterized by an increase in negative tweets and a decrease in both positive and neutral tweets from August 2019 to September 2019.

**Table 1:** Examples of positive, negative, and neutral tweets. Each tweet is assigned to only one of the categories based on its sentiment score calculated by VADER.

Sentiment category	Tweet	Score
Positive (N = 205,338)	@[ID] proud of you! Please dont switch to vaping. The nicotine and stuff in it is way more addictive than cigs. Nicorette (gum) is supposed to be helpful. Others i know who quit just bought lollipops to have something to put in their mouth. Seems to help her [smiling emoji] [heart emoji] [heart emoji] [heart emoji] [heart emoji]	0.9872
	I’m so excited to actually achieve my Quit Smoking goal. I really hope I’m finally over cigarettes [finger-crossed emoji]	0.7083
	Yep. The packaging still looks like a cigarette box, but instead of gum, it’s a stick of white sugar.	0.3291
Negative (N = 107,428)	cravings, restlessness, trouble concentrating, irritability, anxiety. what can i ask more than this? But, going cold turkey is the only way out i think! #QuitSmoking	−0.5848
	My whole family is clumsy. I broke my own toe closing a door because I didn’t pull my foot away in time. (I was sober) Also fell off a roof sneaking a cigarette as a kid & ended up splitting open my head. Still took me 30 years to quit smoking.	−0.4215
	Hard to do. I did it after 25 years because I really wanted to. Went to USA with nicotine gum. Came back a non smoker but was hard. Respect! Now losing the weight too.	−0.088
Neutral (N = 84,762)	I should smoke less	0
	Smoking Cessation Tip #3 [URL]	0
	Delve into the details of @[ID]‘s May 15 public scientific workshop on youth e-cigarette cessation treatment strategies in the latest Update EXTRA: [URL]	0

### Topic modeling

After testing different numbers of topics, we found that the coherence score increased with more topics, peaking at 10 topics with a coherence score of 0.410. Beyond 10 topics, the coherence scores plateaued or declined, indicating diminishing returns in topic quality. The results of LSA, including the ten topics before manual grouping and their respective top 10 words in terms of weights, are presented in S1 Fig.

**Fig 3:**
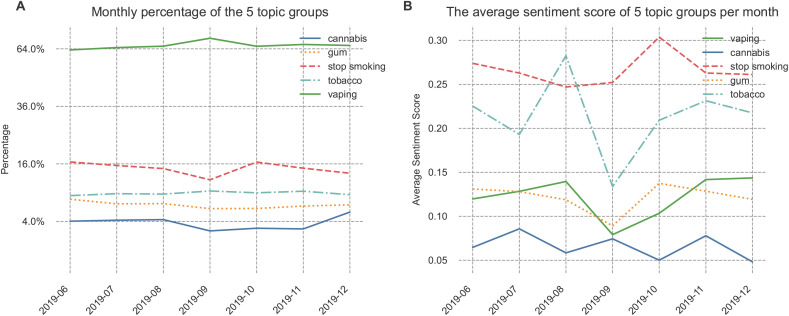
Monthly volume distribution and sentiment score of five topic groups. (A) Volume distribution of 5 topic groups per month (B) The average sentiment score of five topic groups per month.

Table 2 outlines five topic groups, their top 10 aggregated terms, and a summary of representative tweets. The topic groups include “Vaping”, “Cannabis”, “Stop Smoking”, “Gum”, and “Tobacco”. Regarding the monthly volume percentage of each topic group, Fig 3A indicates that discussions on “Vaping” predominated during the observed period, peaking in September 2019. Conversely, “Stop Smoking” and “Cannabis” experienced a decline in volume during September 2019, while “Gum” and “Tobacco” remained relatively stable. Fig 3B presents the sentiment scores for each topic over time. The results reveal that from August 2019 to September 2019, “Tobacco” tweets witnessed the most substantial decline in average sentiment score, dropping from 0.28 to 0.13 (a decrease of 54%). Meanwhile, sentiment scores for “Vaping” and “Gum” also decreased, albeit to a lesser extent, from 0.14 to 0.08 (a decrease of 43%) for “Vaping” and from 0.12 to 0.09 (a reduction of 25%) for “Gum”. The last column of Table 2 provides a summary of representative tweets for each topic group, with detailed representative tweets for each topic presented in S1 Text.

**Table 2:** The theme, top terms, and description of five topic groups.

Theme	Terms	Summary of representative tweets
Vaping (N = 254,437)	vaping, cessation, weed, start, stop, quitsmoking, good, cigarettes, nicotine, smoker	Some expressed concerns about the lack of scientific evidence supporting vaping as a quitting method, although some viewed e-cigarettes as a good way to quit smoking.
Cannabis (N = 15,284)	weed, gum, choice, drink, high, gum disease, man, less than, so much, eat	Tweets labeled “Cannabis” conveyed a variety of attitudes toward smoking marijuana. Some had no intention of quitting. Some tweets deviated from the goal of smoking cessation.
Stop Smoking (N = 68,227)	stop, quitsmoking, cessation, good, vaping, weed, free, tobacco, health, vape	This theme focused on direct appeals to stop smoking, often promoting cessation programs or providing tips for quitting.
Gum (N = 25,042)	start, gum, cigarettes, tobacco, nicotine, smoker, use, vape, chew, new	Tweets on this theme discussed nicotine gum’s effects on quitting smoking. Opinions were mixed, and most thought nicotine gum was helpful. Some believed vaping was at least as effective as chewing gum.
Tobacco (N = 34,538)	cigarettes, tobacco, free, smoker, nicotine, use, support, work, health, free smokeless	The tweets expressed smoking tobacco causes serious health problems. Many supported using aids like caffeine or snus for harm reduction. Some are market promotions.

### Text summarization of Tweets before, during, and after September 2019

Regarding text summarization, for representative tweets before September 2019 (September 2018 – August 2019) in Part 1 in S2 Text, 7 out of 25 (#1-#7) directly focus on vaping, while the others discuss personal experiences of smoking cessation and alternative methods. Among the seven vaping-related tweets, one (#3) mentioned that vaping would not cure nicotine addiction. However, all of these tweets perceived vaping as a method to quit smoking due to its perceived lower health risks. Additionally, one tweet highlighted flavor as a primary reason why people successfully quit smoking using vaping.

S2 Text Part 2 presented the representative tweets in September 2019. During September 2019, 17 out of 25 representative tweets (#1- #17) centered around vaping. The majority conveyed the perception that vaping served as a means to quit smoking compared to other alternatives, with some tweets emphasized the role of e-cigarette flavors in aiding smoking cessation. These tweets exhibited more emotional intensity, characterized by an appealing tone, the use of swear words, and a more direct expression of opposition to restrictions on vaping. Among the 17 vaping-related tweets, four tweets (#4, #9, #11, #17) more explicitly addressed the EVALI outbreak, mentioning e-cigarette use by young people and/or the planned e-cigarette control policies by policymakers. Two tweets (#11, #17) suggested that teenagers’ increased interest in vaping was their fault for misusing e-cigarettes, arguing against blaming e-cigarettes and suggesting that restrictions on e-cigarettes impede smoking cessation. One tweet (#4) recognized vaping as a smoking cessation option but also acknowledged the necessity for policymakers to impose restrictions due to the potential misuse by teenagers.

As illustrated in Part 3 of the S2 Text, following September 2019, 14 out of 25 representative tweets (#1-#14) centered around vaping. The proportion of vaping-related tweets notably increased compared to the period before September 2019 and remained consistent with that observed during September 2019. Many users continued to view vaping, mainly flavored e-cigarettes, as an effective method for smoking cessation, drawing from their personal success stories. The overarching themes observed in these tweets closely resembled those identified in September 2019.

## Discussion

It is the first study to examine the smoking cessation-related discussion and the sentiment trend on Twitter before and after the EVALI outbreak. Through tweets between September 1, 2018, and January 31, 2020, our analysis indicates a rise in the discussion of smoking cessation and a decline in sentiment towards smoking cessation during the EVALI period. Vaping emerges as the predominant topic within smoking cessation discussions.

We found that the overall sentiment score toward smoking cessation was positive, and tweets of all topic groups, including “Vaping”, were also predominantly positive. This result is consistent with the study by Van der Tempel et al. [20]. In their analysis of a human-coded sample of tweets (N = 120,803) related to smoking cessation and vaping from January 1 to December 31, 2014, they found that the sentiment was mostly positive and neutral and advocated using e-cigarettes for smoking cessation.

Additionally, we found that in September 2019, the EVALI outbreak inspired more reflections on smoking cessation and a drop in sentiment scores. This decline was primarily driven by the decreased sentiment in tweets focused on “Vaping”, given its predominance among all topic groups. This result aligns with the study by Kasson et al. [14], which showed a higher proportion of negative tweets in September 2019, concurrent with the heated discussion of vaping.

Furthermore, despite declining sentiment for vaping in September 2019, we observed that the sentiment remained predominantly positive. This finding contrasts with another study that analyzed vaping-related tweets (N = 286,703) from July 2019 to September 2019. The study revealed that Twitter feeds contained more negative posts than positive ones in August and September, resulting in an overall negative sentiment [21]. The disparity in sentiment polarity could be attributed to their focus on discussions solely related to vaping, whereas our study examined vaping within the context of smoking-cessation-related tweets.

Our analysis of representative sentences during and after September 2019 reveals a favorable sentiment among individuals toward flavored e-cigarettes, which aligns with findings from a study by Kirkpatrick et al. [28]. Their research documented a significant public response on Twitter opposing e-cigarette flavor bans during the EVALI outbreak. The phrase “Flavors Save Lives” or hashtag “#FlavorsSaveLives” was frequently employed to express this opposition during EVALI [33].

Essentially, EVALI highlights the potential harm of vaping, which could make people reconsider using e-cigarettes to quit smoking. However, our results indicate a persistent positive sentiment toward vaping for smoking cessation even after EVALI. Many people still view vaping as a helpful tool for quitting smoking and express dissenting reactions toward the regulations on e-cigarettes. This demonstrates the difficulty of influencing public perception. In a landscape where evidence on e-cigarettes’ effectiveness for quitting smoking remains mixed [34], and marketing practices and regulations continue to evolve, it underscores the need for policymakers to develop more effective strategies to deliver clear, evidence-based messages. Furthermore, it is important to communicate FDA-approved pharmacotherapy, such as varenicline, bupropion, and nicotine replacement therapy [35], more clearly to the public. Public health crises like EVALI also present an opportunity to reshape public perceptions, highlighting the importance of learning from EVALI to improve future responses.

Specifically, studies have shown that individuals who struggle with smoking cessation tend to be more active in online discussions and use less favorable language [36]. Therefore, the discussions on smoking cessation may predominantly mirror the perspectives of these individuals who have faced multiple failed attempts to quit smoking. These heavier posters, who have experienced repeated difficulties, are in greatest need of support and assistance in quitting smoking. We should develop communication strategies to address their concerns and misconceptions, ensuring a more targeted and resonant approach to public health communication. Such efforts are crucial for dispelling misconceptions, delivering accurate information, and fostering a comprehensive understanding among the public.

### Limitation

It is important to note that our investigation solely focused on the text contained within tweets. However, it is worth acknowledging that users convey their opinions through text and multimedia elements such as pictures, videos, hyperlinks, and other attributes of tweets like replies, retweets, and likes. These additional dimensions of information also play a significant role in reflecting people’s perceptions and viewpoints. Future studies are warranted to use these non-text tweets to examine people’s perceptions of smoking cessation thoroughly.

Secondly, LSA utilizes word occurrences to summarize topics from texts, making it possible to extract commonly used but less informative words to represent topics. We applied LexRank to extract additional information for each topic to address this limitation.

Furthermore, our keyword selection aimed to balance recall and precision but may have omitted terms relevant to smoking cessation. Expanding the keywords could capture more tweets but might introduce noise, affecting validity. While the demographics of adult e-cigarette users and Twitter users do overlap, with both groups more likely to be younger and more educated than the general population [37,38], Twitter data are not fully representative, thereby, the results of this study should be interpreted with caution.

## Conclusion

Our study identified an increase in smoking cessation discussion and a significant decline in sentiment toward smoking cessation after the EVALI outbreak. Moreover, vaping emerged as the predominant topic of discussion, with sentiments towards vaping also diminishing. Despite the EVALI outbreak, many still perceive vaping as an effective smoking cessation tool. Our result has a broader global implication. Smokers around the world use e-cigarettes to quit smoking. Our results highlight how public perceptions about smoking cessation evolve during a critical health event. The findings emphasize the need for policymakers worldwide to actively engage with social media, tailor communication strategies to promote scientifically accurate information about vaping and smoking cessation.

## Supporting information

S1 Fig Top 10 words and their associated weights for the 10 topics prior to manual grouping. (EPS)

S1 Text Representative tweets of the 5 topic groups. (DOCX)

S2 Text Representative sentences for LexRank before, during, and after September 2019. (DOCX)

S1 Appendix Comparison between VADER and other sentiment analysis models. (DOCX)
