# Supplementary material for: E-cigarette or vaping product use–associated lung injury outbreak and public perceptions and trends in smoking cessation discussions on Twitter
Source: PLoS One. 2025 Sep 18;20(9):e0332414. doi: 10.1371/journal.pone.0332414 (PMC12445456; doi:10.1371/journal.pone.0332414)
Supplement: S1 Text — (DOCX) [file pone.0332414.s002.docx]

# Representative tweets of the 5 topic groups^[[1]](#footnote-1)^

## Vaping

1. "been thinking a lot lately about quitting smoking..i'm thinking maybe i want a new pet to hold me accountable ; instead of paying for cigarettes, i want a beta to pay me not to smoke.. 🤔maybe a vape slave too..?dm if you're interested in helping me quit.• findom •",
2. ' i never said vaping was okay. i would never recommend someone vape if they don’t already smoke or vape. no one should start. but for smokers, it’s a great alternative and a good way to quit smoking. i was clearly referring to those that believe nicotine is the issue.',
3. 'vaping devices have not been proven to help adult smokers quit smoking. however, vaping increases the risk a teen will smoke regular cigarettes later. do we really want the next generation addicted to nicotine? ',
4. 'guess what y’all? i’ve been nicotine and smoke free for 6 months now. actually over. and i don’t vape. vaping is still smoking. so if you have any inkling of wanting to quit. i’m here. it’s hard but you can do it.',
5. 'every cigarette you smoke can take years off your life. it’s never too late to quit. #livesmokefree #quitsmoking #nonsmoker #quitsmokingapp #smokefree #healthylife #healthiswealth #tobacco #smoking #vaping #cigarettes #smokers ',
6. 'more quit smoking tips: #stopsmoking #quit #non #tips #smoker #smoke #cessation #cigarettes #ecigs #ecigarettes #electronic #vape #vaper #vaping #drugs #nicotine #addiction #withdrawal #nrt #patches #zyban #champix #tobacco #tar #replacement ',
7. 'prepare your mind to stop smoking: #stopsmoking #quit #non #tips #smoker #smoke #cessation #cigarettes #ecigs #lobby #ecigarettes #electronic #vape #vaper #vaping #drugs #nicotineaddiction ',
8. 'more quit smoking tips: #stopsmoking #quit #non #tips #smoker #smoke #cessation #cigarettes #ecigs #ecigarettes #electronic #vape #vaper #vaping #drugs... ',
9. '"i stopped smoking in preparation for my future.” ❤️#switchwithpodsalt and start your smoke free journey!🙌get in touch with us direct or visit for more information!#quitsmoking #vape #vaping #help #change #quit #future #stopsmoking ',
10. 'each day that you don’t smoke is a small victory! keep going! #quitsmoking #livesmokefree #nonsmoker #quitsmokingapp #smokefree #healthylife #healthiswealth #tobacco #smoking #vaping #cigarettes #smokers ',

## Cannabis

1. ' quit smoking weed'
2. 'i’m gonna quit smoking weed one day',
3. ' my dad used to deny it alllll the time; then when i knew for sure he’d say he quit; what not lol. once my bf; i got together my bf would always give him weed, and they would smoke together and finally like a year or two ago my dad and him were smoking in the garage and my',
4. 'now that i don’t smoke weed im back to smoking milds 😩 i just need to quit overall 🤷🏾\u200d♀️',
5. "i'm gonna quit smoking weed. and by quitting i just mean paying for it. if you wanna smoke me out, then by all means, bro. let's chief",
6. 'me: "why can't josh gordon just quit smoking weed long enough to make millions and retire, then he can smoke all the weed he wants?" also me: *can't go 3 days in a row without binge eating despite having a literally life-threatening condition that requires losing weight to fix.*',
7. 'most of you know don’t know i also smoke cigs, i need to quit. i’ve smoked for 30 years and my health is affected. i know if i wanna keep smoking weed i need to quit the cigs. so i bought a juul yesterday instead of more cigs. i guess i’m asking you all for support. 🤞i need it.',
8. 'ive been trying to quit smoking weed so i can get a job at the juvie center. its hard when i smoke it to help me with my most of my problems or stress',
9. ' kameltoe’s daddy told her in jamaican, according to reliable sources, “... quit smoking the weed mon. stop being a ho mon. everybody know our bisness now mon. why you smoke; they call you crackhead mon?” kameltoe responded by stuttering and saying “... wish my willie was here.”',
10. ' good s***. i just passed 8 years off prescription pain pills; i’m pretty proud of it too. i quit drinking in january, but i’m still smoking weed and don’t really plan on quitting anytime soon.',

## Stop smoking

1. ' commissioned jbi to assist with revising 2nd edition of ‘supporting smoking cessation: a guide for health professionals’. to develop the guideline we used grade methodology. read revised guide: #gradeapproach #quitsmoking ',
2. "healthy heart tip: quit smoking. if you smoke, it's time to quit. at any age quitting improves your health. there are many options to assist you in quitting and your doctor can help you succeed. more info #quitsmoking #heartmonth #canescares ",
3. 'maybe your 2018 new year’s resolution was to quit smoking? if it was, congratulations on being smoke free for a year! find out more about discounted life insurance for vapers. #vape #quitsmoking ',
4. 'it’s been 8 months since i picked up smoking again since the last 8 months have been crazy. but i made a promise to myself that i would quit this year and start healthier habits. its been 3 days since my last smoke and i hope to never pick up another ciggy again #quitsmoking 🚬',
5. 'do you need extra support to stop smoking for good? can you use your experiences to support others in quitting smoking? check out bella community now! #quitwithbella #community #app #stopsmoking #quitsmoking #health android: ios: ',
6. '“every time i quit, the urge to smoke was so strong that i started smoking again. after countless failures, 2baconil nicotine patches were the only thing that made a difference.” rahul mehta, 2baconiluser and successful quitter #quitsmoking #testimonial #truestory #motivation',
7. 'need help to quit smoking? our dedicated stop smoking adviser can support you to quit for good! give us a call on 01572 725805 to book your appointment today.#quitsmoking #wellbeing #rutland #rcws',
8. 'the smoker lungs app concept makes vivid the harmful consequences of continuing to smoke in a personalized way that is sure to encourage more smokers to quit. #smoker #lungs #smoking #ar #augmentedreality #app #quitsmoking ',
9. " hey, i caped for two years to get off smoking and now i'm off nicotine for two months. the best thing that has helped me is just going for walks! it gets your blood moving and gives you time to clear your head. good luck! #quitvaping #quitsmoking",
10. '‘i need to have a smoke to relax after work.’ sound familiar? the my quit route app can help. use the app’s tips and prompts to rethink your relationship with cigarettes and stop smoking for good. 👉 #quitsmoking #stopsmoking #stopsmokingmedway ',

## Gum

1. ' nicotine is next level addiction, i’ve being trying to quit for years... i don’t smoke cigs but i vape and chew the gum',
2. ' i tried chantix, wellbutrin, nic gum nothing worked. oh i quit for a couple of weeks then i start smoking again. until i found vaping i never smoked another cig again. i wanted to be around for my family.',
3. 'e-cigarettes are twice as effective as nicotine patches or gum at helping people quit smoking traditional cigarettes. take a look at this study: ',
4. ' jimmy, have you tried the gum? i know for me, in the mornings with my coffee is when i want to smoke the worst. the nicotine gum, kept my mouth busy and helped with the cravings. i need to quit smoking also.',
5. 'okay f*** you twitter. i’m sitting here thinking... damn i need to quit smoking and start hitting the gym. and now i’m getting nicorette gum advertisements. this s*** a simulation',
6. " i quit with the gum fter 30 years of smoking but that was in the 80ies. i kept telling myself i'd start smoking again when i turned 82. i'm about there.",
7. ' limit yourself, and find a hard candy that you like and keep gum handy. it’s easier to quit if you go outside to smoke to me because it involves effort.',
8. " i don't smoke, but my cousin who had smoked since 15 quit using nicorette gum. but now she's addicted to the gum! good luck!",
9. '1/4 of americans smoke. the flame retardants in cigarettes can be carcinogenic... and the fires caused without them take lives... the only solution is to quit smoking.. through nicotine patches or gum, hypnosis, willpower',
10. ' do not go down that path. it took me 3 times to quit over 2 years. i used the gum 4mg and the trick was to follow the directions to the letter. i tried the gum once before and it made me smoke more but i using it wrong.',

## Tobacco

1. 'on sale free usa shipping on all 5 packs of cowboys coffee rodeo chew quit chewing\xa0or dipping tobacco nicotine free smokeless smoking alternative to snuff cigarettes ay\x7f30c6anj mlb nfl nhl nba nascar ',
2. 'attending the wales tobacco health network today this morning, which is looking at the question “who can afford to smoke?” many spending more on cigarettes than food and many of those are the poorest in our society. most would like to quit smoking. ',
3. 'what is your take on the tobacco #harmreduction controversy? 1.1 billion on earth people smoke. smoking is the the number one cause of preventable cancer, heart and lung disease. most smokers want to quit. their addiction is now medicalized stigmatized. but... ',
4. 'people who use tobacco twice as likely to get gum disease as someone who doesn’t smoke. smokers are also more at risk for oral cancer. give yourself a gift this valentine’s day and quit today. #azfamilydentistry ',
5. 'have you tried or are you trying to quit smoking cigarettes pipe smoke cigars or chewing tobacco snuff try pinches of caffeine vs nicotine smokeless snus 100% safe for consumption mlb nfl nhl nba nascar ',
6. 'oral mouth cancer are most common in seniors. if you smoke or use tobacco, or regularly drink alcohol, you have an increased risk of developing lung or oral cancer gum disease. contact us about how to quit tobacco and learn more here: #toothwisdom ',
7. 'the risk of #stroke for smokers of tobacco products is twice as high as that of non-smokers. this includes not only cigarettes, but also cigars, pipe tobacco, and chewing tobacco. #askyourpharmacist about ways to help you quit smoking. #heartmonth ',
8. 'it’s day 9 of trying to quit smoking and i’ve had 6 cigarettes all in all. i had 2 on sunday whilst out for a roast which i regret but hey it’s all part of the process. i’ve also run out of tobacco which makes it a lot heard to smoke 🚭',
9. '. says tobacco use is the most preventable cause of death. $2 tax increase will help than 30,000 oregonians to quit smoking. [/](about:blank) smoke shop owners bristle at proposed oregon tobacco tax increase ',
10. '#didyouknow the health problems of smoking are caused by other components in tobacco smoke not by nicotine? nicotine replacement therapy is one of many useful quitting aids you can use to quit for good. for more information visit '

1. All representative tweets are cleaned. [↑](#footnote-ref-1)
