# Supplementary material for: E-cigarette or vaping product use–associated lung injury outbreak and public perceptions and trends in smoking cessation discussions on Twitter
Source: PLoS One. 2025 Sep 18;20(9):e0332414. doi: 10.1371/journal.pone.0332414 (PMC12445456; doi:10.1371/journal.pone.0332414)
Supplement: S2 Text — (DOCX) [file pone.0332414.s003.docx]

# Representative sentences for LexRank before, during, and after September 2019

## Representative sentences before September 2019^[[1]](#footnote-1)^

1. 'vaping helped father quit smoking. missouri smoke free encourages and support the use of vapor products to aid in quit attempts. #quitsmoking #newyearsresolution2019 #ecig',
2. " it helped me stop smoking after 30 years. the last 15 of which was a solid 2 pack a day habit. i had tried everything to quit. obviously putting anything in you're lungs is not ideal. 2.5 years tobacco free and i go weeks now without vaping. only vape when urge to smoke is strong",
3. ' i agree with how you put that, "less bad." yes, vaping is less bad than smoking because of decreased exposures to toxins in smoke. however, vaping with nicotine does not cure a nicotine addiction. it can help someone quit, the same way nicotine gum can. but it is still nicotine.',
4. " yes they do, but far less than cigarettes do. nobody should start smoking e cigs, but they are a great alternative for nicotine addicts who wish not to smoke tobacco. regulate them. stop pretending they aren't better. who supports them as an alternative.",
5. ' every smoker is different. no under-age person should vape or smoke. but some smokers start vaping and "quit by accident" the next day. a lot actually. others are dual users for months, but eventually quit smoking. vaping replicates behavior (it's not just about nicotine).',
6. ' regardless of what his job is...i vape menthol and another friend smokes reg tobacco flavor..all my nieces who used to smoke and now vape do the fruity flavors and have quit smoking..i’m speaking from personal experience..adults like flavors, sweets and fruity tastes',
7. 'quit smoking cigarettes, vaping is the new trend! #vapelovers #ecigars #vape #vaper #bongs #bong #vapeproducts #juul #juullover #amazing #great #wonderful #smoke #flavors #blowclouds #cloud #eliquid #ecigarette #electroniccigarette #bongvape #l4l #canada #granvillevape '
8. 'do you smoke? do you want to quit? fact: you’re four times more likely to kick the habit if you use a stop smoking product and get advice or support – simply click here for help: #quitsmoking #antismoking #selfhelp #quickmist #healthyyou #health #fitness',
9. 'so you want to stop smoking? #stopsmoking #quit #non #tips #smoker #smoke #cessation #cigarettes #ecigs #lobby #ecigarettes #electronic #vape #vaper #vaping #drugs #nicotineaddiction ',
10. "i quit smoking cold turkey. it's now 5 days minus an hour i'm smoke free. i've been a 2 pack/day for 32 years. imagine how good it is to be a non smoker ; use it as my power base for quitting. when craving comes, i acknowledge it's my addiction talking #quitsmoking #coldturkey",
11. 'quitting #smoking is really tough. #tooth decay, gum disease and oral #cancer are all possibilities when you smoke or use #tobacco, so work with your #dentist and doctor to get the support you need to help quit smoking. ',
12. ' i never quit. i stopped smoking this took away the stress of sneaking in a cigarette. i smoked a few packs after stopping. i also chewed a lot of gum and that helped. it comes down to having a good reason to stop and sticking by that reason when you want to smoke.',
13. 'tobacco cravings are temporary. to stay quit, find ways to get through the 5-10 minutes you want tobacco the most join us as we fight for a #smokefree world koşar adim #ar public health #game #tabac #fumar #smoke #smoking #smokingkills #cigfree #quitsmoking#gamedesign #gamedev ',
14. '#mondaymotivation so ... yesterday @ 11:45 am i quit smoking ✊ this is not a new years resolution it is just me simply not wanting to smoke anymore 🤷\u200d♀️ i am using the patch chewing gum and my xanax the xanax is the smallest dose possible 0.25 😂 but it works i have terrible',
15. ' i quit but my husband still smokes. it was a little harder with him smoking. but he does not smoke in the house. i also did it with just using trident cinnamon gum and winter-o-green lifesavers. it’s been over two years since i quit.',
16. 'have you tried or are you trying to quit smoking cigarettes pipe smoke cigars or chewing tobacco snuff try pinches of caffeine vs nicotine smokeless snus 100% safe for consumption mlb nfl nhl nba nascar ',
17. "new year's resolution to quit smoking? start by dropping the chai and smoke combo. naye saal mein karo shuruat, #ekcigarettekam ke saath!try nicotex gum and quit smoking in 12 weeks with the who-approved nicotine replacement therapy. to know more visit- ",
18. 'quit smoking easily with nicomeltz! its anti-smoking nicotine strips melts instantly in your mouth and calms your smoke craving in seconds.order now: fast delivery | authentic products | vast product range #knotss #tabletshablet #nosmoking #quitsmoking ',
19. 'smoking: do you let a poisonous habit dictate your day? there are 4000 poisons in a cigarette - how much do you need to know to quit and be around for longer? 07875720623#smoke #smoking #quitsmoking #stopsmoking ',
20. 'more smoking cessation tips: #stopsmoking #quit #non #tips #smoker #smoke #cessation #cigarettes #ecigs #ecigarettes #electronic #vape #vaper #vaping... ',
21. 'have you tried or are you trying to quit smoking cigarettes pipe smoke cigars or chewing tobacco snuff try pinches of caffeine vs nicotine smokeless snus 100% safe for consumption mlb nfl nhl nba nascar ',
22. 'this is james! james smoked 3 packs a day, and decided to #quitsmoking after having an asthma attack. he tried a cigarette a year after he quit and was so sick that he threw up. now, james says he won’t smoke again and encourages others in his life to slow down or quit smoking. ',
23. 'quit smoking in cardiff: #stopsmoking #quitsmoking #non #tips #smoker #smoke #cessation #cigarettes #ecigs #ecigarettes #electronic #vape #vaper #vaping #drugs #nicotine #addiction ',
24. '30pcs natural auricular therapy magnet stop smoking patches quit smoke plaster smoking cessation nicotine patch cigarettes() ',
25. 'have you tried or are you trying to quit smoking cigarettes pipe smoke cigars or chewing tobacco snuff try pinches of caffeine vs nicotine smokeless snus 100% safe for consumption mlb nfl nhl nba nascar ',

## Representative sentences during September 2019

1. ' i used to smoke but now vape and chew. i want to quit it all for health reasons. at the same time, i hate thinking about all the money that i flush down the drain on it esp vaping. between the e-juice and replacement coils it is more expensive than smoking.',
2. 'people: "i don't even smoke anymore. i quit! i only vape now."vaping. is. not. safe. vaping is not some healthy alternative to smoking cigarettes. i've seen vaping advertised in that manner. that's not true.you can get sick from #vaping. it is not harmless: ',
3. ' good for you jim i quit smoking with lemon drops by candy king i smoke for more than 30 years and tried gum, patch and pills only thing work for me was vaping',
4. " i'm a cigarette smoker that is trying to quit. i now use a vape. i agree minors should not be vaping or smoking. i smoke menthol cigs and that's the type of vape liquid i use. would the ban on flavors keep me from getting my menthol? keep up the good work.",
5. "if you're trying to quit nicotine, and vape, i do not recommend using any pens that use nicotine salts. the levels in them are much higher than i thought...even higher than cigarettes. smoking a cigarette gets me no buzz at all and the urge to smoke right after a cig is real!",
6. " i was a 30 year two pack a day smoker. i had a heart attack at 42. i tried all cessation methods available. the pills gave me chest pain, i'm allergic to the patch, the gum made me want to smoke. vaping was the only thing that helped me to quit. for that reason",
7. "well, i have friends who successfully quit smoking by switching to vaping, but it may be that nicotine gum or the patch is still safer. what's most important, if you don't smoke, please don't start. be safe, be healthy ❤️❤️❤️ ",
8. ' no. using vaping to quit smoking is like using nicotine patches or gum to quit smoking the problem with alcoholic drinks is the alcohol the problem with smoking is the smoke, not the nicotine nicotine deliver via vapor is likely much less damaging to your health than combustion',
9. ' what about the parents who smoke ;want to see their kids; grandkids grow up. taking away the most effective way to quit smoking(which a study just concluded vaping was the most effective way to help quit smoking then the gum,patch,; meds combined) isn’t the way to go about it.',
10. ' if you no longer smoke cigarettes, you have quit smoking. if someone switches to the gum or patch, which contain same nicotine, have they not quit? the rate of decline in smoking rates have sped up since vaping was introduced. that is 10 million ex smokers.',
11. 'these f****** kids is vaping cuz it’s kool. these idiots don’t know why people use it. people who use to smoke use them to quit smoking like myself. #quitsmoking i’m slowing going down on my nicotine amount so i can completely quit nicotine and smoking for good #nosmoking',
12. 'tried everything to quit smoking , patches , gum ,inhalator even government stop smoking program, only thing that worked ... innokin coolfire 4 and blueberry menthol e liquid , 4 years smoke free and feel grrreat!',
13. " i successfully used vaping to quit smoking after 25 years of smoking cigarettes, and then was able to use vaping to wean myself off of nicotine altogether. i am now vape and smoke free. i may not have been able to ever quit smoking if i didn't have vape as a step down alternative",
14. 'people turn to vaping to quit smoking, slowly reducing their nicotine levels through the use of e-liquids. the benefit of e-cigarettes, compared to regular cigarettes, is that they do not produce the tar or the toxic gases found in cigarette smoke. ',
15. " vaping helped me quit smoking. i have tried the patch, read allan carrs book, chewed the gum. i quit several times ; always went back to cigarettes. vaping flavors is a cleaner feeling, no ashes, no second hand smoke, no littering. i am a bus driver ; don't come back with stink ",
16. "i started to smoke at the age of 7 years old. i've tried everything, the gum, patches, and cold turky. you name it i've tried it. then i found a watermelon pod system. i would of never quit smoking if it was menthol or tobacco flavor.",
17. 'vaping is meant to be a short term thing to help smokers quit smoking, not for kids to use for three years. this is not the majority, however if you are a young kid using vape products like juul because it’s “trendy” or you want nicotine but don’t want to smoke,it’s your fault. '
18. "get ready for #stoptober a day early! with smoke free you'll have all the best tools you need to quit smoking ❌🚬 scoring a huge 92% on orcha! see our review here: #smokefree #quitsmoking #mhealth #orcha",
19. " sells hoodies that you can smoke vape out of the string. that makes it easier to hide smoking. it doesn't help anyone quit smoking dangerous cigerettes.",
20. 'here are reasons that i can’t justify myself smoking cigarettes:-my grandpa (dad’s dad) smoked all his life and quit when i was born, he later died of lung cancer -my dad is battling his second time w throat cancer, fairly likely due to second hand smoke exposure as a child',
21. " tbh i can't smoke normal cigarettes. i don't like the overpowering tobacco. if capri went out of business i'd probably quit smoking. everyone gives me s*** for them but when they need to bum one they always praise how smooth they are",
22. ' if they no longer smoke cigarettes, they have quit smoking. if someone uses the gum or patch, which contains same exact nicotine, but not longer smoke, they too have quit smoking.',
23. 'how can people quit somoking, if the nictine patch and gum are $40 or more a pack each? i do not smoke but geez that seems pretty high for the goal to be to reduce smoking.',
24. " try sunflower seeds or chewing gum. helped me out tons when i quit smoking. i've also seen some people replace smoking/vaping with physical activity for the duration it would take to smoke every time they would normally smoke",
25. ' people who break their addiction to smoking tobacco by replacing cigarettes with nrt (patches, gum etc) are not addicted to nicotine. they are nicotine dependent. do i care about nicotine dependence in the absence of tobacco smoke? no i do not. yours, a smoking cessation expert.',

## Representative sentences after September 2019

1. ' i quit smoking cigarettes using flavored vapor products. 5 years smoke free after smoking cigarettes for 15 years. my lungs feel amazing and i have my life back thanks to vaping.',
2. " funny how nicotine patches and gum didn't work for me. chantix made me want to commit suicide. only vaping flavored ejuice helped me quit smoking. 3 years smoke free!",
3. " i've tried the gum, patches and pills and it didn't work very well. i tried to quit smoking with vaping back in 2009 and it didn't work either but, tried again this past march. haven't smoke a cigarette in over 6 months. i think the flavors are much better now then in 2009 imo.",
4. ' fda approved cessation methods dont always work. i tried patches, gum, chantix and none of it helped, even when i developed a terrible cough... but with vaping i quit right away and my cough was gone within weeks! 6 years smoke free now',
5. " once you taste the sweet sweet mango nicotine, you'll never smoke the cancer sticks again.my pops quit this way in a week, after smoking for 50 years. he tried every smoking cessation method under the sun, nothing worked until vaping. that pisses johnson;johnson and gsk off.",
6. ' i agree. i’ve smoke since preteen, have quit, ; restarted multiple times. i am addicted to nicotine. when my neighbor was diagnosed w/ lung cancer, her doctor recommended vaping to stop smoking cigarettes. she and i are both cigarette free. my bp, kidney, lung health improved',
7. ' life saving for 6 ppl i know ~quit smoking via vaping. my pcp suggests vaping to ppl to kick the habit. a vape shop tapers you down to zero nicotine. i strongly suggest weighing to pros for many adults smoke free.',
8. 'ivva take great exception to statement in this article that we are trying to addict young people to nicotine. ivva members have helped many thousands of irish smokers become smoke free. vaping is most popular quit smoking tool in ireland. ',
9. ' 15yrs pack a day smoker of newports and quit with the help of vaping flavored nicotine and i have been smoke free for 7-8 yrs. i tried to quit smoking cigarettes with nicotine gum, patches, and medication nothing worked until i tried vaping.',
10. 'freakonomics "for smokers trying to quit, vaping offers an experience that nicotine patches and gum don’t: “there's physical stimuli… the throat hit, seeing the smoke come off. there's social stimuli, smoking with other people in social settings.” — … "',
11. "for smokers trying to quit, vaping offers an experience that nicotine patches and gum don’t: “there's physical stimuli… the throat hit, seeing the smoke come off. there's social stimuli, smoking with other people in social settings.” — professor ",
12. ' you earned yourself a follow sir, dr. 😊 harm reduction saves lives. no one is perfect, but for those that smoke, or did smoker (quit via nicotine vaping product) ends are a godsend and the gateway away from deadly tobacco. 95% safer than continued smoking.',
13. "vaping doesn't help adults quit smoking is a lie. been smoke free for 3 years from a 20+ year of tobacco cigarettes. strawberries and cream e-liquid from my local vape shop is and continuing to save my life #iquitsmokingwithvapor #wevapewevote",
14. 'i am a 36 year old who smoked for 15 years. i tried to quit with nicotine patches, gum, and medications. nothing worked then i found out about vaping which helped me quit smoking cigarettes for good. now i am smoke and nicotine free. vaping is 95% safer. harm reduction.'
15. ' breaking the habit part is the hardest but most successful way to quit.. also i have mints and gum packed in my car because i always want to smoke while driving. 7 years smoke free after 20yrs of smoking',
16. 'do you or did you #smoke after most meals? for many who just quit #smoking, the end of a meal can be a trigger. try brushing your teeth after a meal or chewing gum, or doing something to keep your mind and hands busy. 😁 ',
17. ' i quit a year ago today!! i didn’t smoke for nearly as long but i was smoking for about 8 years. i did it cold turkey because i didn’t want to pick up a new habit in gum or vaping and the first 3 weeks were the worst of my life but i’m so happy i stuck it out. you got this!!',
18. " yay! it'll get easier ; easier over time. i quit years ago ; i never, ever think about smoking or wanting a cigarette. i did initially chew on straws, ; sunflower seeds (; maybe gum). i also started working out regularly, running, ; eating better which helped me not want to smoke",
19. 'have you tried or are you trying to quit smoking cigarettes pipe smoke cigars or chewing tobacco snuff try pinches of caffeine vs nicotine smokeless snus 100% safe for consumption mlb nfl nhl nba nascar ',
20. 'have you tried or are you trying to quit smoking cigarettes pipe smoke cigars or chewing tobacco snuff try pinches of caffeine vs nicotine smokeless snus 100% safe for consumption mlb nfl nhl nba nascar ',
21. 'today i celebrate 1 year free of nicotine.seen is the 7200 cigarettes i would have smoked this year 🚬 .photo credit 📸:#smokefree #smoking #quitsmoking #tobacco #cigarette #cigarettefree #smoke #quitsmoke #cigarettes #wellness #healthy #healthyliving #change ',
22. 'have you tried or are you trying to quit smoking cigarettes pipe smoke cigars or chewing tobacco snuff try pinches of caffeine vs nicotine smokeless snus 100% safe for consumption mlb nfl nhl nba nascar ',
23. ' to quit smoking she chose to smoke something other than cigarettes? that doesn’t seem productive. maybe gum or candy, but doing something that’s basically the same doesn’t seem smart',
24. 'i’m trying to quit smoking for good this time, i only had about a pack and a half of cigarettes for 6 days, which is good for me. i’m using the nicotine lozenges, they work better than the gum. i’m really getting bad urges to smoke, nicotine is like heroin to me. ',
25. ' quit smoking cigs 11 years ago, cold turkey. first 3 days were somewhat hard. then suddenly things tasted better ; i could suddenly smell how horrible cig smoke really smells. i washed everything that smelled like smoke, chewed a lot of gum ; mints. best thing i ever did! do it!',

1. All tweets are cleaned. Vaping-related tweets are in bold. Tweets responding to the EVALI outbreak are highlighted. [↑](#footnote-ref-1)
