# Supplementary material for: E-cigarette or vaping product use–associated lung injury outbreak and public perceptions and trends in smoking cessation discussions on Twitter
Source: PLoS One. 2025 Sep 18;20(9):e0332414. doi: 10.1371/journal.pone.0332414 (PMC12445456; doi:10.1371/journal.pone.0332414)
Supplement: S1 Appendix — (DOCX) [file pone.0332414.s004.docx]

# Comparison between VADER and other sentiment analysis models

We compared VADER’s sentiment scores with three models: TextBlob (rule-based), EmoBart (deep learning), and Twitter-roBERTa-base (deep learning).

To effectively compare the algorithms, we randomly selected 10,000 tweets from our dataset and employed different sentiment intensity models to label them. As there were no ground-truth labels for these tweets, we calculated the correlation between their sentiment intensity scores, indicating consistency across models.

In addition to VADER, we selected another rule-based model - TextBlob Pattern[7] and two deep learning models: Twitter-roBERTa-base from TweetEval [8] and Emobart-large from EmoLLMs [9]. Each model has a different approach to sentiment scoring: TextBlob provides sentiment intensity between -1 and 1, EmoBart outputs a sentiment intensity from 0 to 1, while Twitter-roBERTa-base assigns discrete sentiment labels (0 for negative, 1 for neutral, and 2 for positive). For the VADER vs. RoBERTa comparison, we used the percentage of mutual agreement rather than correlation, as RoBERTa outputs categorical labels.

The table below shows the correlation coefficients between the models’ sentiment scores:

| Model Comparison | Correlation/Agreement |
| --- | --- |
| VADER vs. TextBlob | 0.5074 |
| VADER vs. EmoBart | 0.4927 |
| VADER vs. RoBERTa | 0.5389 |

This analysis reveals moderate correlations between VADER and the other models, with the strongest alignment observed between VADER and RoBERTa. The moderate correlation demonstrates that VADER provides results comparable to other models, despite being rule-based and trained on different datasets. Given VADER’s interpretability and consistency with more complex models, it remains a suitable choice for analyzing sentiment within our dataset.
